# Editorial: Growth regulators and biostimulants: upcoming opportunities

**DOI:** 10.3389/fpls.2023.1209499

**Published:** 2023-05-26

**Authors:** Shubhpriya Gupta, Paromik Bhattacharyya, Manoj G. Kulkarni, Karel Doležal

**Affiliations:** ^1^ Laboratory of Growth Regulators, Faculty of Science, Palacký University & Institute of Experimental Botany Academy of Sciences of the Czech Republic (AS CR), Olomouc, Czechia; ^2^ Research Centre for Plant Growth and Development, School of Life Sciences, University of KwaZulu Natal Pietermaritzburg, Scottsville, South Africa; ^3^ Biotechnology Division, Council of Scientific and Industrial Research-Institute of Himalayan Bioresource Technology, Palampur, India; ^4^ Department of Chemical Biology, Faculty of Science, Palacký University, Olomouc, Czechia

**Keywords:** plant growth regulators, biostimulants, soil health, organic, sustainable agriculture, crop productivity

Plant growth regulators (PGR) and biostimulants are known to regulate plant growth and development, boost plant metabolism, and enhance nutrient uptake, resulting in healthier plants with better yields. However, their mode of action to promote plant growth is different. Plant growth regulators are defined as synthetic compounds, phytohormone derivatives such as [e.g., 2, 4-Dichlorophenoxy Acetic acid), alpha-Naphthalene Acetic Acid, 6-benzylaminopurine, thidiazuron and 6-furfurylaminopurine or kinetin] that mimic naturally occurring plant hormones [e.g., indole-3-acetic acid, *trans*-Zeatin, *cis*-zeatin, dihydrozeatin DZ, isopentenyladenine] (Gianfagna, 1995; Scacchi et al., 2009; Gupta and Chaturvedi, 2022). Growth regulators do not hold nutritive value and function by either suppressing or boosting plant growth and development by directly influencing plant hormones at low doses (Rademacher, 2015). On the other hand, biostimulants usually are complex mixtures containing organic (example, extracts of seaweed, vermicompost leachate, protein hydrolysates, humic substances, smoke-water), microbial (fungi and bacteria) and/or inorganic (Si, Se) (Brown and Saa, 2015; Colla and Rouphael, 2015; Du Jardin, 2015; Gupta et al., 2021; Shahrajabian et al., 2023) constituents. They enhance plant growth and health by stimulating natural processes at a minute quantity rather than directly controlling plant growth (Rouphael and Colla, 2018; EBIC, 2020).

Plant growth regulators are required in low quantities and have fewer impurities than conventional fertilizers. Furthermore, biostimulants are derived from natural resources (Gupta and Van Staden, 2021). Therefore, there is a minimal risk regarding toxicity and safety to humans and the environment (Kisvarga et al., 2022). Growth regulators and biostimulants are becoming increasingly popular among farmers and consumers worldwide because they can help increase yields and improve soil health with low dependence on synthetic fertilizers and pesticides. There is a paradigm shift around the world post COVID-19 and an increase in demand for organic food among consumers as a preventive health measure both from developed and developing countries (Ćirić et al., 2020; Śmiglak-Krajewska and Wojciechowska-Solis, 2021; Brata et al., 2022; Wang et al., 2022). With a growing global population and increasing concerns about environmental degradation there is a growing demand for sustainable agriculture practices and the adoption of GLOBALGAP (GLOBAL Good Agricultural Practices) policies (Kleemann et al., 2014; Mook and Overdevest, 2021). Furthermore, the market for growth regulators and biostimulants is expected to grow and expand into new areas, including Asia and Africa (Markets and Markets, 2022; Markets and Markets, 2023). Growth regulators and biostimulants are likely to become more significant in agriculture over the next few years due to technological advancements and growing demand for sustainable agriculture.

In 2022, PGR and biostimulants market was valued at circa (i.e., approx., ca.) USD 2.9 billion and ca. USD 3.5 billion respectively (Markets and Markets, 2022; Markets and Markets, 2023). For PGR, it is projected to reach ca. USD 4.5 billion with a CAGR (Compound Annual Growth Rate) of 7.4% by 2028 (Markets and Markets, 2023) and ca. USD 6.2 billion and 11.8% for biostimulants by 2027 (Markets and Markets, 2022). Therefore, both small and big companies like, Isagro (Italy), Arysta (Japan), BASF (Germany), Syngenta (Switzerland), Bio AG Alliance (US), FMC Corporation (US), Valagro (Italy), Kelpak (South Africa), Biolchim (Italy), Acadian (Canada), Koppert (Netherlands), Biostadt (India), Italpollina (Italy) and many more are pushing into growth regulators and biostimulants and are significantly investing in research (Corsi et al., 2022, Markets and Markets, 2022; Critchley et al., 2021; Moyo et al., 2021). With the growing popularity of growth regulators and biostimulants, many countries are developing regulations to ensure the safety and effectiveness of these products (Du Jardin, 2015; García-Sánchez et al., 2022). Furthermore, as regulatory frameworks advance, there will be opportunities for PGR and biostimulants companies to develop innovative products that satisfy regulatory requirements and fulfill the needs of farmers (Caradonia et al., 2019; Norrie et al., 2021).

Recent technological developments have enabled more efficient growth regulators and biostimulants to be developed. For example, the application of nanotechnology can enhance the efficacy and delivery of these products suggesting that growth regulators and biostimulants will become more significant in agribusiness in the coming years. The research papers in the focus Research Topic on “*Growth Regulators and Biostimulants: Upcoming Opportunities*” highlight the importance of growth regulators and biostimulants in agriculture and their role in improving crop yield in sustainably under biotic and abiotic stress. In this Research Topic, Rathore and Kumar studied the dynamics of phosphorus and biostimulants [Amino Booster G (the amino acid solution) and V-Hume (the humic acid solution)] on the agro-morphological traits, essential oil yield, and chemical constituents of German chamomile (*Matricaria chamomilla*). They found that the amino acid and humic acid solution positively affects plant growth, flower yield, and essential oil composition. Li et al. discovered that exogenous application of trehalose to maize (*Zea mays*) generated more significant carbon and nitrogen metabolic activity, increased chlorophyll content, and enhanced dry matter accumulation in roots and shoots as compared to the effect of chitosan, humic acid and gamma-aminobutyric acid. Parmar et al. reviewed the metabolites produced by microalgae biostimulants and their effects on plant growth, productivity, and tolerance against stressors, as well as different modes of application of microalgae metabolites. Furthermore, the authors emphasized the circular economy model of microalgae-mediated bioremediation coupled with biorefinery approaches for generating high-value metabolites and enhancing the sustainability of microalgae biomass production. Mohammed et al. demonstrated that the seed presoaking and irrigation of broad bean (*Vicia faba*) and sunflower (*Helianthus annuus*) with *Sargassum polycystum* aqueous extract improved the germination, growth and antioxidant activity of plants. Raj et al. discovered that biostimulants like vermicompost, biofertilizer, and liquid seaweed extract enhanced basil (*Ocimum basilicum*) yield and quality without using harmful agrochemicals.

Wang et al. explained the molecular mechanisms of melatonin and its function in promoting adventitious root formation in cucumber (*Cucumis sativus*) seedlings grown under shade. The authors found that melatonin significantly increased adventitious root formation in the cucumber hypocotyl by controlling the expression of genes involved in hormone synthesis, signaling, and cell wall biogenesis, as well as by raising levels of auxin, cytokinin, jasmonic acid, salicylic acid, and abscisic acid.

Plant growth regulators can be used to alleviate the detrimental effects of biotic and abiotic stress and improve crop yield and quality. Singh et al. showed that various hormones (such as ABA, cytokinin, GA, and IAA) differently control flowering in saffron by regulating floral integrator [*FT* (*FLOWERING LOCUS T*) and *LFY* (LEAFY)], repressor [*SVP* (*SHORT VEGETATIVE PHASE*) and *TFL1-2* (*TERMINAL FLOWER1*)] genes, and homeotic [*PISTILLATA*, *SEPETALLA*, and *DL* (*DROPPING LEAF*)] gene expression. Otari et al. found that MS medium fortified with 2.0 mg/l BAP + KIN and 0.5 mg/l IAA + IBA + NAA produced the best shoot and root development results, respectively, in Indian Pennywort (*Bacopa floribunda*). The role of cytokinin in defense or stress priming and the maintenance of photosynthesis was meticulously reviewed by Hudeček et al.. Debnath and Ghosh reviewed in-depth study on the phenotypic variation in micropropagated berry plants and the role of DNA methylation in these variations. Pandey et al. showed that foliar spray or seed soaking treatment of a novel natural plant growth enhancer, “calliterpenone,” (a phyllocladane diterpenoid) isolated from *Callicarpa macrophylla* enhanced crop productivity of wheat (*Triticum aestivum*), rice (*Oryza sativa*) potato (*Solanum tuberosum*), chickpea (*Cicer arietinum*), tomato (*Solanum lycopersicum*), and onion (*Allium cepa*).

In this Research Topic, the study by Zhang et al. found that Ca^2+^-treated pears can suppress the production of stone cells which affects pear quality, and provided insight into its molecular mechanism. Liu et al. discovered that among carbon dioxide, ethylene, nitrogen, and wounding, carbon dioxide treatment was the best to induce the formation of heartwood in 6-year-old Indian sandalwood (*Santalum album*) trees.

Kaushal et al. reviewed the molecular pathways activated by microbial biostimulants (plant growth-promoting rhizobacteria, PGPR) in plants facing abiotic and biotic stress. Morcillo et al. reviewed the effect of cell-free microbial culture filtrates on beneficial soil microbes, plant growth promotion and in combating stress tolerance. Ali et al. reported that the application of stress-tolerant *Bacillus* sp. improved the length of shoots, roots, and number of roots in saffron (*Crocus sativus*). del-Canto et al. showed that the inoculation of common bean (*Phaseolus vulgaris*) with drought and salinity-tolerant indigenous rhizobium strains improved drought tolerance and yield compared to nitrogen fertilization.

Plant growth regulators and biostimulants have great potential to improve agricultural yield, making them a great option for farmers and growers looking to increase productivity and profitability in a sustainable manner. The use of plant growth regulators and biostimulants can help reduce the environmental impact of chemical fertilizers by increasing nutrient uptake, improving soil health and fertilizer management practices. With advancements in technology and growing demand for sustainable agriculture, there are numerous opportunities for PGR and biostimulants. For example, precision agriculture techniques can be used to improve the efficiency and effectiveness of PGR and biostimulants. These techniques can aid in boosting crop quality and yield. In addition, the use of biostimulants and PGR is spreading beyond conventional crops like grains and vegetables to include ornamental, medicinal, and fruit-producing plants. Plant growth regulators and biostimulants will likely play a crucial role in improving the productivity of crops and minimizing environmental impact by prioritizing sustainable agricultural practices.

## Author contributions

All authors listed have made a substantial, direct, and intellectual contribution to the work and approved it for publication.
